# Ranking places in attributed temporal urban mobility networks

**DOI:** 10.1371/journal.pone.0239319

**Published:** 2020-10-14

**Authors:** Mirco Nanni, Leandro Tortosa, José F. Vicent, Gevorg Yeghikyan

**Affiliations:** 1 Department of Computer Science and Artificial Intelligence, University of Alicante, Alicante, Spain; 2 Institute of Information Science and Technologies, Italian National Research Council, Pisa, Italy; 3 Scuola Normale Superiore, University of Pisa, Pisa, Italy; UNSW, AUSTRALIA

## Abstract

Drawing on the recent advances in complex network theory, urban mobility flow patterns, typically encoded as origin-destination (*OD*) matrices, can be represented as weighted directed graphs, with nodes denoting city locations and weighted edges the number of trips between them. Such a graph can further be augmented by node attributes denoting the various socio-economic characteristics at a particular location in the city. In this paper, we study the spatio-temporal characteristics of “hotspots” of different types of socio-economic activities as characterized by recently developed attribute-augmented network centrality measures within the urban *OD* network. The workflow of the proposed paper comprises the construction of temporal *OD* networks using two custom data sets on urban mobility in Rome and London, the addition of socio-economic activity attributes to the *OD* network nodes, the computation of network centrality measures, the identification of “hotspots” and, finally, the visualization and analysis of measures of their spatio-temporal heterogeneity. Our results show structural similarities and distinctions between the spatial patterns of different types of human activity in the two cities. Our approach produces simple indicators thus opening up opportunities for practitioners to develop tools for real-time monitoring and visualization of interactions between mobility and economic activity in cities.

## Introduction

The ever-growing availability of large scale data sources pertaining to human activities in contemporary cities and the fact that the socio-economic and technological systems lend themselves adequately to representation through discrete elements and interactions between them have led recent years to witness an unprecedented increase in modelling of such complex systems using network theory [1].

In urban science, there has been a significant research interest towards understanding urban systems particularly through modelling road structures, human mobility, traffic flow, and economic activity through a complex networks approach [2–4]. In such a setting, distinct elements in a city such as road junctions or neighbourhoods are typically represented as the network nodes, while the heterogeneous connections or interactions between them, such as road segments, passenger flows, activity correlations represent the edges in the network [5, 6]. Further, depending on the focus of the research, various statistical and graph-theoretical properties of the network can be studied to gain valuable insights about the urban spatial, temporal and socio-economic structures. Following this approach, several studies have analysed mobile phone usage, taxi or private car GPS trajectories, smart card, geo-located social media, and classical census data for inferring systemic patterns both at the individual and aggregate level [7–11].

An area of research of particular interest in complex network theory is the study of the importance of nodes or edges in a network through centrality measures. Such measures are typically based on local and global network connectivity structures and include a variety of types: degree [12], closeness [13], betweenness [14], eigenvector [15], PageRank [16], etc. However, these conventional centrality metrics measure the importance of nodes by considering only the network topology regardless of the intrinsic information on these nodes such as their behaviour, type or some other, domain-specific attribute. Since many kinds of real-world networks call for such node attributes, several centrality measures have recently been proposed extending the widely used centrality measures to accommodate node attributes [17–19]. This becomes especially relevant in urban modelling, as locations in a city possess quantitative and qualitative characteristics irrespective of the connectivity structure of the network of interactions with other locations. Such characteristics may describe the availability and quantity of such urban features as parking lots, restaurants, real estate prices, population density, etc., qualitatively enhancing urban networks.

Another important line of research in complex networks is temporal network theory: the study of the evolution and behaviour of networks over time. Temporal networks integrate network science with time-series analysis and contribute greatly to the modelling of epidemic spreading, transportation optimization, biological systems, as well as social networks [20].

Although some recent work has focused on analysing the spatial patterns of different urban features [5, 21], studying urban networks with centrality measures [17, 22, 23], as well as modelling the evolution of urban interaction networks over time [24], we still have a poor understanding of the interplay between urban location characteristics and the networks of interactions between these locations. All the more so, the temporal evolution of this interplay remains an unexplored area of research.

Having this gap as motivation, the objectives of this paper are to analyse and study the spatial distribution of the central nodes by activity type over time in urban origin-destination (*OD*) networks. More specifically, we focus on the spatial arrangement of the most central nodes of the *OD* network as identified by the Adapted PageRank Algorithm (APA) [17] additionally considering activity related to food and retail services over time in Rome and London. We find that although the daily temporal patterns of the most central places in attributed *OD* flows in the two cities display structural similarity, the spatial distributions of food and retail related activity over time differ, indicating a more polycentric structure in London. The proposed pipeline from raw GPS and open source point-of-interest (PoI) data to the resulting data visualization offers a workflow with the potential for creating tools for monitoring the changes in mobility patterns and in their relations to various socio-economic activities over time. This would allow urban practitioners to monitor daily/weekly mobility patterns for analysing the effects of urban interventions or temporary events, but also to study long-term trends in these patterns for urban policy making.

To achieve the objectives the structure of this paper is as follows: the theoretical tools employing graph theoretical methods for characterising centrality (Adapted PageRank Algorithm), a measure of statistical heterogeneity (the Gini coefficient) for describing the distribution of the obtained centrality values, a non-parametric technique for identifying “hotspots” of high centrality values, and a spreading index characterising the spatial spread of the “hotspots” in the two cities are presented in *Previous Work* Section. *The data set and methodology* Section describes the dataset used for the proposed study and summarises the methodology underpinning the experiments. The proposed methodology is validated and the numerical results from studying real urban *OD* networks in London and Rome are discussed in *Numerical Results* Section. Finally, *Conclusion* Section 5 concludes the paper.

### Related work

The city is one of the most complex dynamic anthropogenic systems. To analyse this complexity, spatial networks have been widely used for modelling city objects and the interactions between them, and different approaches have been proposed with regards to the choice of objects and the various types of interactions between them [1, 22, 25]. In modelling cities with these simple mathematical objects called graphs, a variety of properties such as the relative importance of city locations through network centrality measures can further be studied.

Network centrality measures have been used in different problem settings across many research fields related to economic geography [26], road networks [22], and urban mobility [23]. [26] study the impact of social network structures exemplified by central nodes computed with the PageRank algorithm in the US startup mobility networks on the innovation performance of cities.

In studying street networks, [22, 25], for instance, analyse the distributions of various types of centrality measures computed on the street networks of different cities and find them to reveal the distinction between self-organized and planned cities. Another work [27] utilises betweenness centrality measures in street networks across cities worldwide to find universal bimodal betweenness regimes corresponding to trees and loops explaining high and low centrality values, respectively. Similarly, conventional centrality measures have also been used in studying human mobility, particularly on inter- and intra-urban *OD* networks. In particular, [28] reveal node betweenness centrality in an inter-urban *OD* network displaying a positive correlation with population and wealth, while [23] study the statistical properties of betweenness centrality in intra-urban *OD* networks in different cities.

Conventional centrality measures suffer from the drawback of not taking account of exogenous information on the nodes. In this regard, there exist studies that have attempted to overcome this by extending centrality measures to include node attributes. [17, 18] propose a centrality measure based on the PageRank to study the key areas of city activity on the street network enriched with geo-referenced retail and services data on the nodes. [29] take a different approach, introducing distance decay and attractiveness modifications to the PageRank algorithm to incorporate the effects of distance and attractiveness in choosing a particular destination over another.

Computing measures of network centrality gives us the relative importance of the nodes (locations) in an urban network. However, choosing the *most* important locations requires some discussion. In the field of spatial analysis, a “hotspot” usually refers to a location with an attribute value relatively higher than that of its neighbouring locations. The study of the spatial characteristics of “hotspots” has been the focus of research in such different fields as criminology [30], transportation [31], or epidemiology [32]. In the context of urban mobility, “hotspots” may be seen to reflect travel intensity between different areas [33, 34]. With the availability of large data streams of ever more granular location data, “hotspot” analysis is becoming a widely practiced tool in urban mobility research [35, 36]. There exist many techniques for urban “hotspot” detection. The first is based on spatial statistical analysis, particularly on spatial autocorrelation indicators for detecting neighbouring areas with dissimilar value intensities [37]. Another “hotspot” detection method is based on kernel density estimation by using a spatial search method [38]. In [39], the authors have applied this method to study the spatial distribution of popular locations. In the context of urban mobility *OD* networks, a “hotspot” detection technique of particular interest has been presented in [40] and further applied in [41], in which the authors develop a method borrowing from economics to construct a Lorenz curve and based on the choice of a threshold exploiting the intuition that the more skewed the distribution of values of interest in a city, the less “hotspots” there will be. We will discuss this method in detail in the *Identifying the hotspots* Section as part of our work. After “hotspot” detection following the described approach, the authors in [40] and [41] study the coarse grained mobility patterns in a city by breaking down the urban mobility OD matrix into a 2 × 2 block matrix corresponding to combinations between “hotspot” and “non-hotspot” locations. The authors in [42] also study and discover universal coarse grained mobility patterns in cities, but instead of using a threshold-based approach, they resort to a non-parametric clustering method for identifying “hotspots”.

So far, we have discussed static networks as the object of study with tools from network theory. However, since many real-world phenomena require modelling their behaviour over time, temporal network theory has become a valuable tool in many fields. This is the case with urban mobility which demonstrates important temporal patterns, the study of which could greatly inform urban planning, policy making, and management. A number of studies has attempted to analyze urban mobility from a temporal perspective. [43] use centrality measures for temporal prediction on *OD* networks built from cellular traffic data. [24], study temporal *OD* networks with change detection techniques for identifying “change points” in time, in which the entire structure of the graph changes.

There have also been recent applications of graph neural networks on temporal sequences of graphs, mostly in a prediction setting. For instance, based on the length of prediction windows, previous studies of traffic forecast can be divided in dynamical modelling [44] based on mathematical tools and physical knowledge, and data-driven methods [45, 46] based on classical statistical and machine learning.

## Previous work

In this section, the centrality measure applied to rank the attributed nodes in the *OD* networks, statistical dispersion measures describing the centrality value distributions, as well as a measure of spatial spread are presented in detail.

### The Adapted PageRank algorithm (APA)

The *PageRank* model [47] was proposed to compute a ranking for every Web page based on the graph of the Web. The objective is the calculation of a vector (*PageRank vector*) which establishes a ranking of all the pages analyzed according to their importance.

The PageRank vector is the dominant eigenvector of the matrix known as *Google matrix* *G*′ (see [16] for an algebraic definition and characteristics of this matrix). This matrix is a stochastic square matrix with non-negative elements and the sum of the elements in each column being equal to unity. It introduces a parameter *α* such that 0 < *α* < 1 known as *damping factor*. This parameter represents the probability that a random surfer in the Web jumps from a page to any other in the network.

Among its spectral features, *G*′ is stochastic and positive, so it can be directly applied the Perron-Frobenius theorem to assure the existence and uniqueness of the PageRank vector $\vec{x}$. To delve into the characteristics of the PageRank model, see [48, 49].

In 2012, [17] proposed an adaptation of the original PageRank model called Adapted PageRank Algorithm (APA) for spatial networks with data, although the original algorithm was initially thought for urban street networks. Afterwards, the APA model was modified introducing variants [18]. The base of the APA model is the construction of an stochastic and positive matrix *M*_*APA*_ that keeps the spectral properties of the Google matrix. Then, it is possible to compute a unique eigenvector that constitutes the classification of the nodes according to their importance in the network.

As the Google matrix had two terms, one related to the node’s connections and the other related to the probability of surfing among the pages, the matrix *M*_*APA*_ has two terms, the first related to the connectivity and the second term related to the data associated to every node. So, a data matrix *D* of size *n* × *k* is constructed where the rows are the nodes and the columns are the attributes of the node’s information object of the analysis.

Therefore, *M*_*APA*_ is constructed from the adjacency matrix *A* and the data matrix *D* as

$$\begin{array}{c} M_{APA}=\left(1-\alpha \right)P+\alpha V, \end{array}$$ (1)

where *P* is the probability matrix computed from the adjacency matrix, and *V* is a matrix that collects the whole data associated to the nodes. Regarding the probability matrix *P*, it is constructed from the adjacency matrix *A*, as

$$\begin{array}{c} \begin{array}{cc} p_{ij}=\{\begin{array}{cc} \frac{1}{c_{j}} & \text{if}\;a_{ij}\neq 0,\\ 0 & \text{otherwise}, \end{array} & 1\leq i,j\leq n, \end{array} \end{array}$$ (2)

where *c*_*j*_ represents the sum of the *j*-th column of the adjacency matrix.

Remark that *P* has the following characteristics: it is nonnegative and stochastic by columns. See [17] to know more details about the spectral properties of *P*.

The parameter *α* introduced in 1 determines the importance we attach to the data within the calculation of centrality, since the matrix *V* is responsible for collecting data on the network. Just as in the Google matrix, the damping factor allowed us to evaluate the possibility that a random navigator would go to any node in the network, in the Adapted PageRank algorithm, the parameter *α* allows us to decide the greater or lesser importance of the data compared to the own connectivity of network nodes. Following the definition of Google matrix, parameter *α* is also defined between 0 and 1, in order to make the *M*_*APA*_ matrix stochastic by columns.

The APA algorithm proposed by the authors can be summarized as:

**Algorithm 1**: APA algorithm for computing the node’s centrality.

**Input**: Let *G* = (*V*, *E*) be a graph representing a network with *n* nodes, let be *D* the data matrix associated to nodes of *G* and let be $\vec{v_{0}}$ the weighted vector.

**Output**: $\vec{x}$ representing ranking of the nodes in the graph *G*

**begin**

 Compute the matrix *P* from the graph *G* according to (2)

 Compute $\vec{v}=D{\vec{v}}_{0}$

 Normalize $\vec{v}$, and denote it as ${\vec{v}}^{*}$

 Construct *V* as $V={\vec{v}}^{*}{\vec{e}}^{T}$

 Construct matrix *M*_*APA*_ following the expression (1)

 Compute the eigenvector $\vec{x}$ of the matrix *M*_*APA*_ associated to eigenvalue λ = 1

**end**

Vector $\vec{x}$ constitutes the Adapted PageRank vector and provides a classification or ranking of the network nodes according to both the connectivity and the presence of data.

### Gini coefficients

After computing the node rankings with the APA centrality for each activity type for each hour of the day, we need measures of heterogeneity to assess their distributions in time and space.

The first type of measure commonly used to assess how heterogeneous a variable is distributed, is the Gini coefficient, borrowed from economics. It is defined as

$$\begin{array}{c} GI=\frac{\sum_{i=1}^{n}\sum_{j=1}^{n}|{\vec{x}}_{i}-{\vec{x}}_{j}|}{2n^{2}\bar{\vec{x}}}, \end{array}$$ (3)

where ${\vec{x}}_{i}$ is the APA value at location *i* = [1, 2, …, *n*] and $\bar{\vec{x}}=\left(1/n\right)\sum_{i}{\vec{x}}_{i}$.

The Gini coefficient, originally used to measure wealth and income inequality, can be applied to quantify the heterogeneity of other variables as well. In the case of characterising heterogeneity of values at different locations in a city, the Gini coefficient will take on the value of zero if the variable of interest is distributed uniformly across city locations. Conversely, it takes on its maximum value when all of the variables of interest are concentrated in a single location, leading to a Gini coefficient of *GI* = 1 − 1/*n*, which is very close to 1 for large *n*.

However, being a measure of statistical dispersion, the Gini coefficient is agnostic to the spatial arrangement of the APA values in the city. As demonstrated in [50] and [51], a reshuffling of the spatial configuration can yield the exact same Gini coefficient.

In order to obtain a Gini coefficient that carries meaningful spatial information, we further use the Spatial Gini index proposed in [50]. In essence, it is a decomposition of the classical Gini with the aim of considering the joint effects of inequality and spatial autocorrelation. More specifically, it exploits the fact that the sum of all pairwise differences can be decomposed into sums of geographical neighbors and non-neighbours:

$$\begin{array}{c} GI=\frac{\sum_{i=1}^{n}\sum_{j=1}^{n}s_{i,j}^{A}|{\vec{x}}_{i}-{\vec{x}}_{j}|}{2n^{2}\bar{\vec{x}}}+\\ +\frac{\sum_{i=1}^{n}\sum_{j=1}^{n}(1-s_{i,j}^{A})|{\vec{x}}_{i}-{\vec{x}}_{j}|}{2n^{2}\bar{\vec{x}}}, \end{array}$$ (4)

where $s_{i,j}^{A}$ is an element of the geographic spatial adjacency matrix.

The Gini index can be interpreted as follows: as the positive spatial autocorrelation increases, the second term in 4 increases relative to the first, since geographically adjacent values will tend to take on similar values. On the contrary, negative spatial autocorrelation will cause an opposite decomposition, since the difference between non-neighbours will tend to be less than that between geographical neighbours. In either case, this offers the possibility to quantify the contributions of these two terms. The results obtained from this approach can be tested for statistical significance by using random spatial permutations to obtain a sampling distribution under the null hypothesis that the APA variate is randomly distributed in space.

### Spreading index

Despite their informative relevance, the Gini coefficient and its spatial variant exploit the mean $\bar{\vec{x}}$, which, under fat-tailed distributions, as many socio-economic variables tend to be, may be undefined. In such cases, as shown in [52], the Gini coefficient cannot be reliably estimated with non-parametric methods and will result in a downward bias emerging under fat tails.

Another downside of measuring heterogeneity of the obtained APA values with the Gini approach is that it does not offer the possibility to study the spatial arrangement of the “hotspots”—locations with very large APA values. The “hotspots” are defined as the grid cells with an APA value above a certain threshold ${\vec{x}}^{*}$ (see Fig 5). For choosing this threshold we resort to a non-parametric method introduced in [40]. Once we have identified the “hotspots” as cells with APA values larger than the chosen threshold ${\vec{x}}^{*}$, we can use the *spreading index* introduced in [51] for measuring the average distance between the “hotspots”, normalized by the average city distance to enable cross-city comparisons:

$$\begin{array}{c} \eta ({\vec{x}}^{*})=\frac{\frac{1}{N({\vec{x}}^{*})}\sum_{i,j}d\left(i,j\right)1_{\left({\vec{x}}_{i}>{\vec{x}}^{*}\right)}1_{\left({\vec{x}}_{j}>{\vec{x}}^{*}\right)}}{\frac{1}{N}\sum_{i,j}d\left(i,j\right)}, \end{array}$$ (5)

where $N({\vec{x}}^{*})$ is the number of pairwise distances of grid cells with an APA value greater than ${\vec{x}}^{*}$, *N* is the number of pairwise distances between all grid cells covering the city, *d*(*i*, *j*) is the distance between cell *i* and cell *j*, and $1_{\left({\vec{x}}_{i}>{\vec{x}}^{*}\right)}$ is the indicator function for identifying the cells with APA values greater than ${\vec{x}}^{*}$ for computing the distances. The *spreading index* is essentially the average distance between cells with ${\vec{x}}_{i}>{\vec{x}}^{*}$, normalized by the average distance between all city cells. If the cells with large APA values are spread around across the city, this ratio will be large. Conversely, if the high value cells are concentrated close to each other, as in a monocentric city, this ratio will be small.

## The data set and methodology

This paper studies the spatio-temporal characteristics of urban mobility in Rome and London, two of the most emblematic and active cities in the world, both from socio-economic and touristic points of view.

The workflow of building the data sets is as follows:

1. The urban territory has been subdivided into *n* Cartesian grid cells of size 1 × 1 km, and each such quadratic cell is considered a node in the graph.
2. The raw GPS trajectories of around 10000 private cars spanning two years have been obtained from proprietary car insurance data for research purposes. The data have been cleaned, processed, and superimposed on the grid. Then, trip origin and destination GPS positions have been identified by interpreting the engine ignition on/off interval for each vehicle. Time intervals between 10 and 35 minutes showed robust outcomes, and 20 minutes were chosen for identifying car trips. The extracted origin-destination points are then mapped to the respective grid cells.
3. The *OD* networks have been built from the extracted origin-destination pairs described by the weighted adjacency matrix, $W^{A}\in R^{n\times n}$, the element $W_{ij}^{A}$ of which represents the number of car trips starting at node (cell) *i* and ending at node *j*.
4. Data matrix $D\in R^{n\times p}$ contains the data associated with each node referring to economic activity related to shopping activity and food-services. It contains three columns that summarize this data, so *p* = 3. The first column collects the number of car displacements from each node to the other nodes. The second column collects the data related to the shopping or commercial activity of each node. In this column we have added the following attributes: shops and shopping malls. The third column summarizes the data referring to the food-services economic sector. We have added the following attributes: restaurants, fast food, bars, cafes. All the features corresponding to food and retail activity per cell has been extracted from geo-referenced data from OpenStreetMap [53].

All the data used in this study can be consulted in the following doi: https://figshare.com/s/d61592a380dd508102b2.

**Fig 1:**
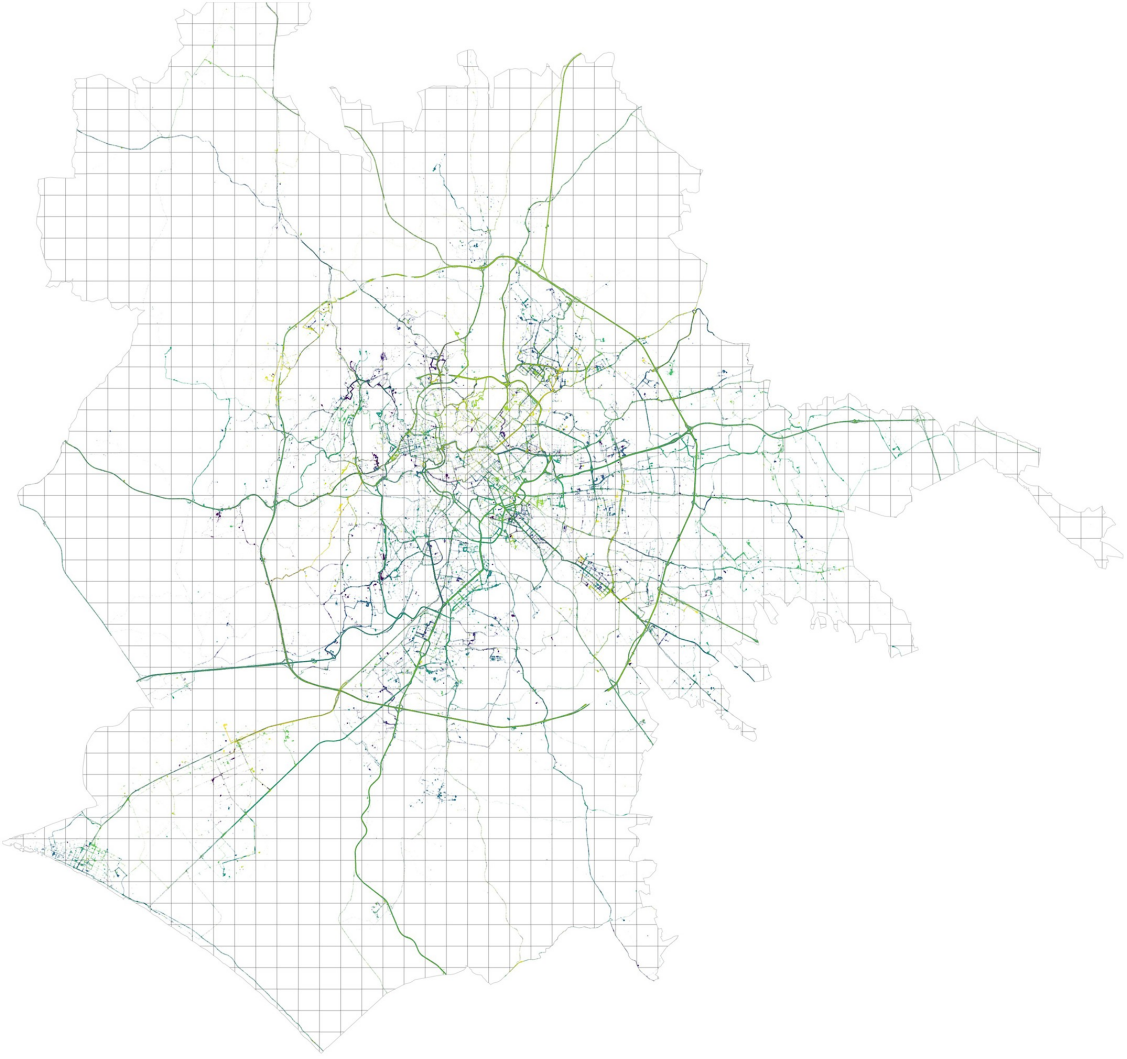
Car GPS trajectories over 1 × 1 km cells in Rome.

**Fig 2:**
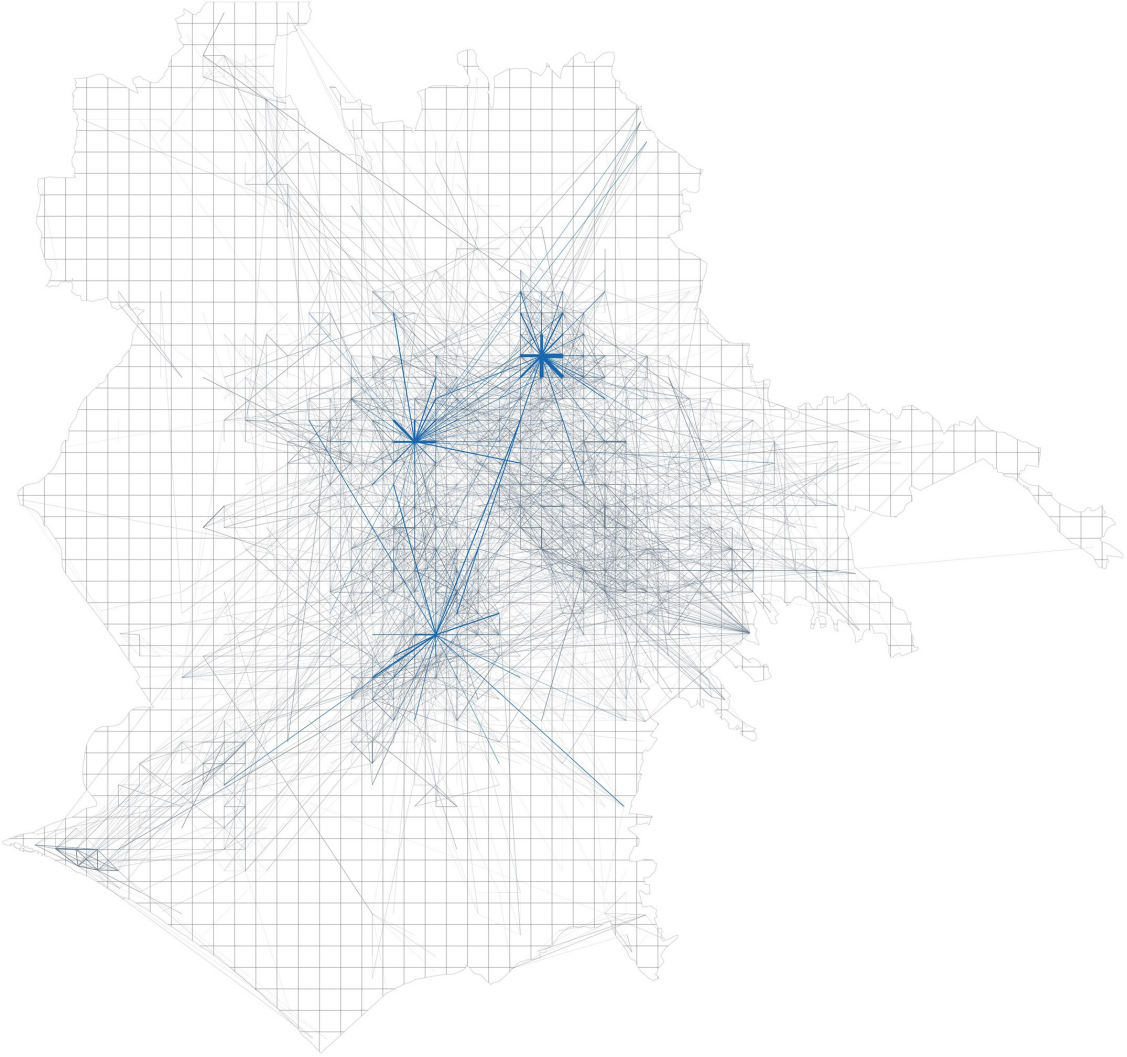
Origin-destination (*OD*) flow network in Rome with some popular travel locations highlighted.

These datasets of a temporal sequence of *OD* networks (see Figs 1 and 2) on a typical day undergo further analysis described in detail in the sections below. We then publicly release a custom data set of location centralities over time obtained from the temporal *OD* flows mentioned above, augmented with node attribute data describing food and retail services activity in city locations.

**Fig 3:**
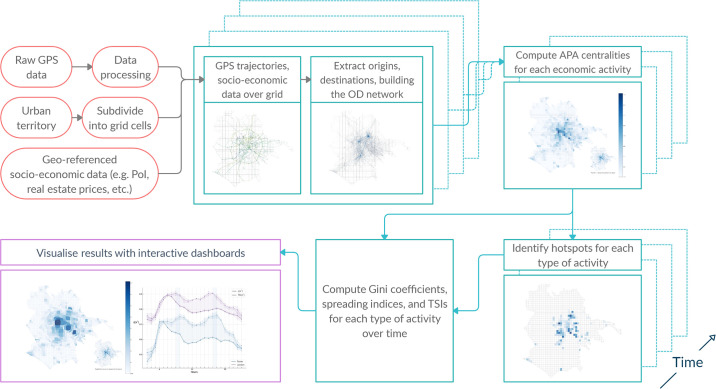
Flowchart of the methodology.

After the city territories have been tessellated into 1x1km grid cells, the raw GPS data has been processed, trip origins and destinations have been extracted and the *OD* networks have been built for each hour of the day both in London and Rome, we proceed to computing the location centralities with the Adapted PageRank Algorithm. Then, we analyze the heterogeneity of the APA values in both cities during a typical day and during a typical week by using the Gini coefficient. Finally, in order to obtain a clearer picture of the spatial distribution of the APA values, we calculate the spreading index and its modification introduced in *The time-space* spreading index (TSI) subsection. The methodology can be summarized in Fig 3.

## Numerical results

In this section we conduct the numerical experiments for the study and outline the principal findings. We then undertake a detailed discussion of the results in the forthcoming section.

### Computing the APA centrality

We proceed to compute the APA values using Algorithm 1 for the following three kinds of networks:

1. Mobility flow network only.
2. Flow network with nodes attributed with information related to retail (shops, shopping malls, retail stores).
3. Flow network with nodes attributed with information related to food services (bars, restaurants, cafes).

Note that for each of these networks we use the corresponding data column of matrix **D** that is in accordance with the economic activity being evaluated.

**Fig 4:**
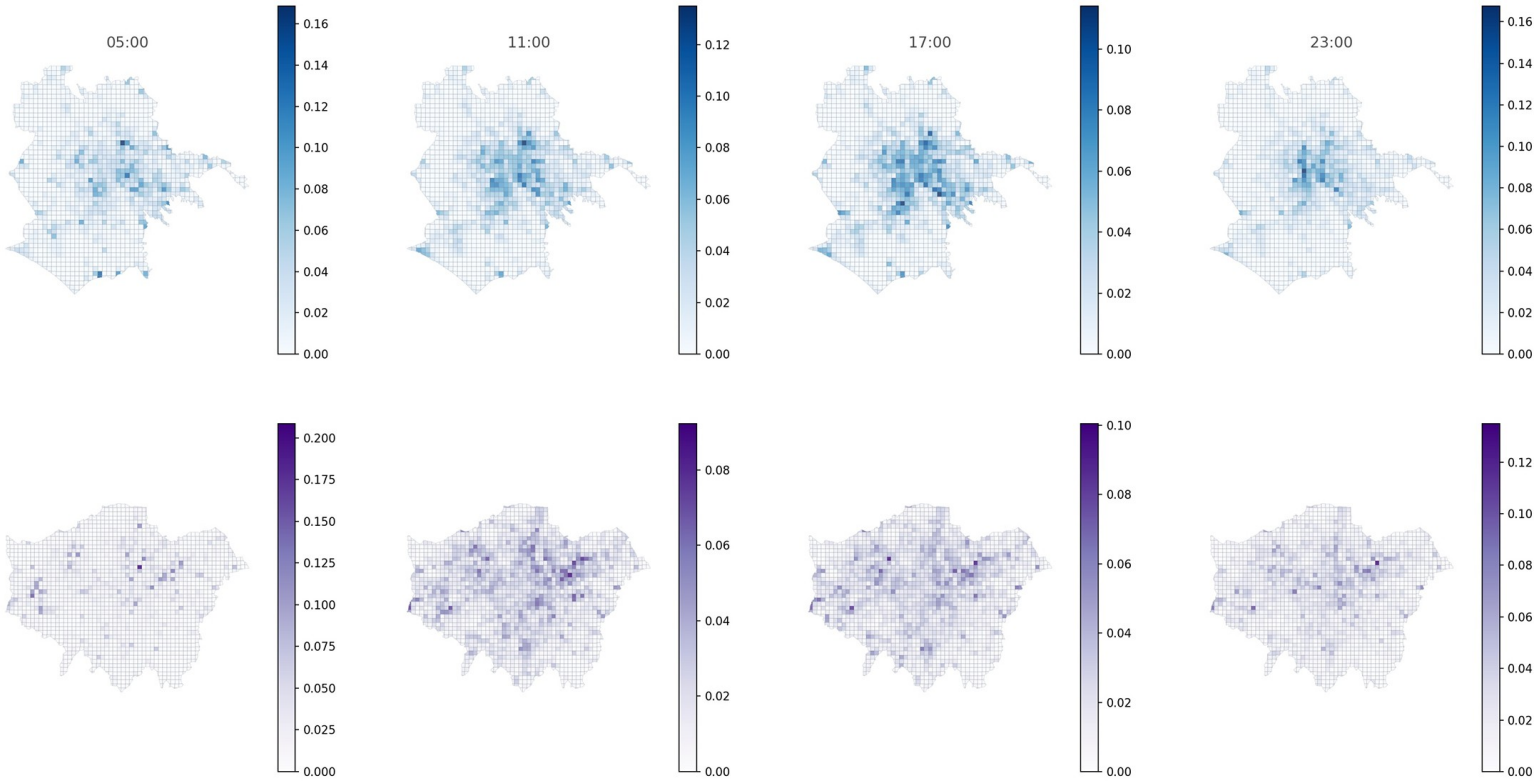
The APA values for the mobility flow network in Rome (up row) and London (down row) at different times of the day.

The APA values of the Rome and London grid cells at different times of the day can be seen in Fig 4. In this Figure, the values of the APA centrality of each of the nodes with respect to the mobility flows have been calculated using Algorithm 1. In the upper row the most central nodes in the city of Rome are clearly shown, at different times of the day; in the lower row the same calculations made in London are shown. Without delving into details, for now, a greater concentration of the most important nodes in the city of Rome is observed for all the chosen times, while in London the most central nodes are in much more dispersed locations. Precisely the study of this dispersion and the characteristics associated with the distribution of centrality values will be one of the axes of this work.

**Fig 5:**
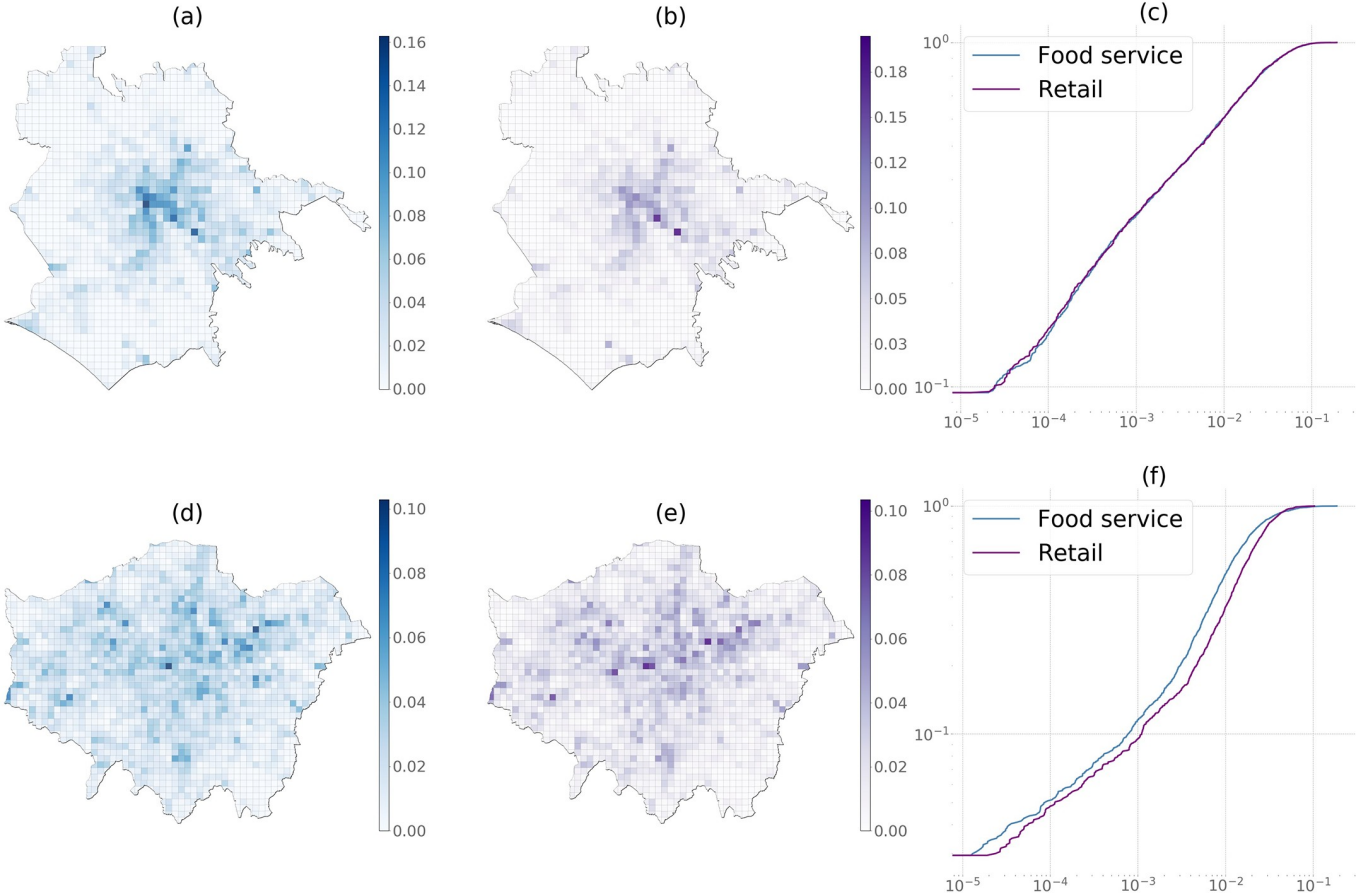
(a)-(b) Food service and retail activity APA distributions in Rome, (d)-(e) in London, (c)-(f) Log-log plots of empirical ECDFs in Rome and London at 12:00pm.

The spatial as well as empirical cumulative distributions (ECDF) of the computed APA values in Rome and London are presented in Fig 5. As can be seen, the APA distributions in both cities are asymmetrically distributed: most of the cells have a very low centrality value, while only a handful of cells have a large centrality value. However, experiments aimed at identifying the analytical distributions yielded different results in the two cities. We conducted the fitting with the Python package “powerlaw” [54]. Parameters obtained via maximum likelihood estimation and the statistical goodness-of-fit measures show differing results for the two cities: a truncated power law distribution for Rome (*p* = 0.004), and a log-normal-like distribution in the case of London (*p* = 0.06). Although the exact distribution is irrelevant here, this finding suggests that different data-generating mechanisms might be in place in the two cities.

### Computing the Gini coefficients

**Fig 6:**
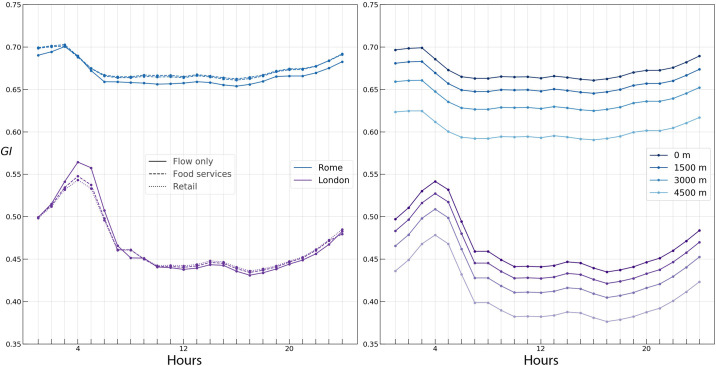
Gini (left) and Spatial Gini (right) coefficients during the day for flow only, food service, and retail activity in Rome and London.

We now proceed to analyzing the heterogeneity of the APA values in both cities, as described in *Gini Coefficients* subsection. In particular, as it is shown in Fig 6 (left), the daily average Gini coefficients in Rome and London take on values roughly 0.67 and 0.48, respectively. The temporal variation of the data is higher in London. In the same Figure, we further observe a slightly higher Gini coefficient during the night hours in both cities, in accordance with the fact that most flows are associated with much fewer areas and thus yield a larger degree of concentration of activity during these hours.

**Fig 7:**
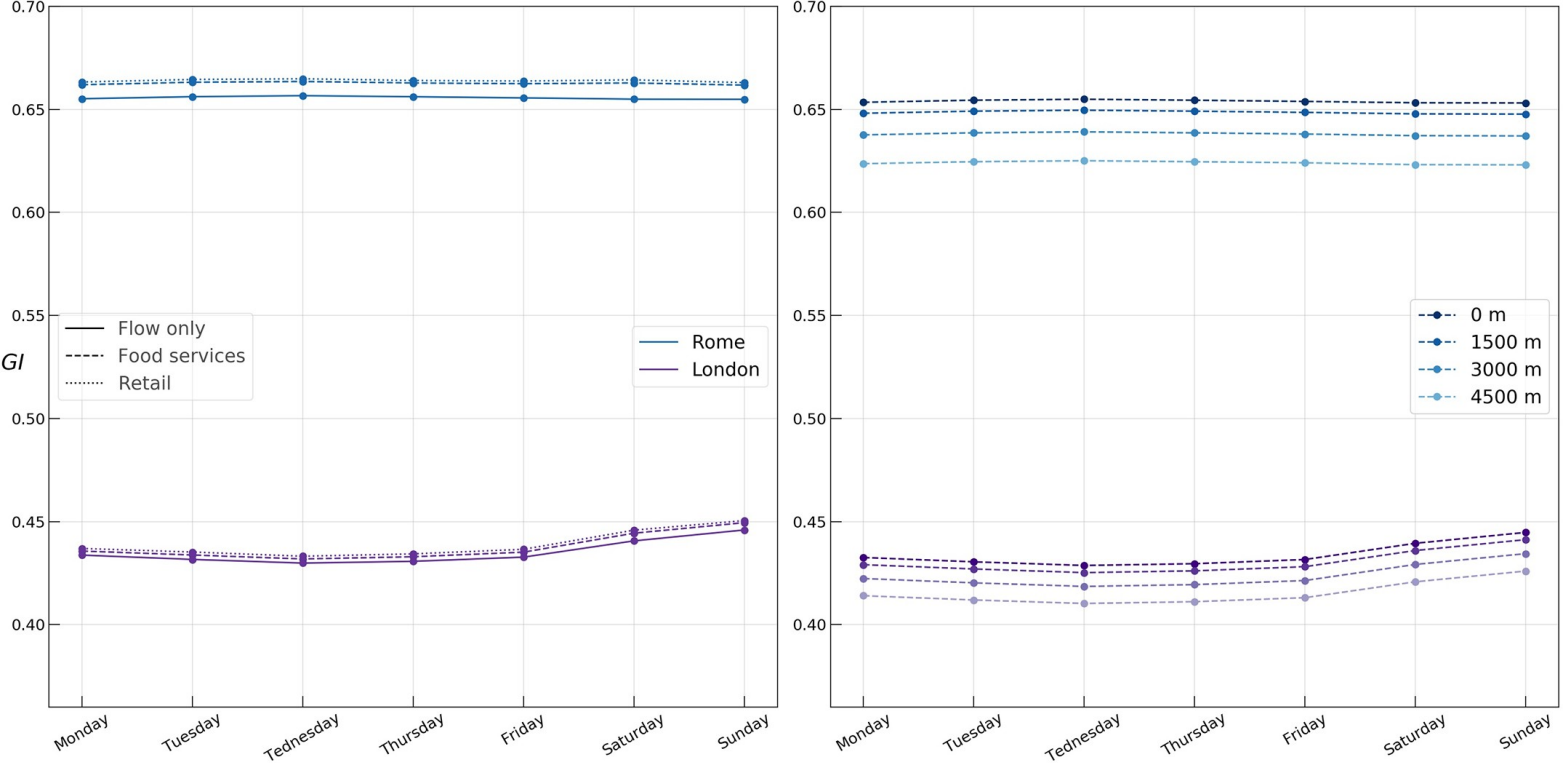
Gini (left) and Spatial Gini (right) coefficients during the week for flow only, food service, and retail activity in Rome and London.

With the aim of finding whether the Gini and Spatial Gini coefficients capture any difference between working days and weekends, both coefficients computed daily are represented in Fig 7. No significant change across the days of the week can be observed neither in Rome nor in London, while only a negligible rise of the coefficient on the weekend can be seen in London.

Despite the fact that some conclusions can be drawn from observing a relatively higher Gini coefficient during the night hours in both cities and on the weekends in London, the temporal evolution of the Gini coefficient, as can be seen in Figs 6 and 7, conveys little significant information. Also, as mentioned in *Gini Coefficients* subsection, it tells us nothing about the spatial distribution of the APA values.

In order to understand the temporal behaviour of the spatial component of the Gini coefficient, we resort to decomposing the Gini coefficient as described in *Gini Coefficients* subsection. In essence, we are interested in finding how much of the Gini coefficient is due to non-neighbour heterogeneity. To achieve this, we follow the approach described in [50] and use the non-neighbour term in the Gini decomposition as a statistic to test for spatial autocorrelation:

$$\begin{array}{c} GI_{2}=\frac{\sum_{i=1}^{n}\sum_{j=1}^{n}(1-s_{i,j}^{A})|{\vec{x}}_{i}-{\vec{x}}_{j}|}{2n^{2}\bar{\vec{x}}}. \end{array}$$ (6)

The expression (6) can be interpreted as the portion of overall heterogeneity associated with non-neighbour pair of grid cells. Inference on this statistic is carried out by computing a pseudo p-value by comparing the *GI*_2_ obtained from the observed data to the distribution of *GI*_2_ values obtained from random spatial permutations. It should be noted that this inference based on random spatial permutations is on the spatial decomposition of the Gini coefficient given by the expression (4), and not the value of the Gini coefficient itself.

Following the described approach, we proceed to the numerical experiments, varying the neighbourhood radius in the expression (6) from 1.5 to 6 kilometers. Both in Rome and London, the random spatial permutation approach yielded a statistically significant spatial decomposition for all hours of the day (p = 0.01). As demonstrated in Figs 6 and 7, the temporal profiles of the Spatial Gini coefficients closely follow the Gini profile. As the neighbourhood radius increases, the inequality due to non-neighbour APA values decreases, since the growing neighbourhood captures more and more of the inequality. We find a superlinear growth in the rate of decline of the Spatial Gini coefficient with increasing the neighbourhood radius, with a faster decline in Rome, suggesting a higher spatial concentration of urban flow in Rome.

### Identifying the hotspots

In order to obtain a clearer picture of the spatial structure of the “hotspot” cells with high APA values over time, we aim to compute the *spreading index* for flow, food services, and retail activity at different hours of the day in both Rome and London.

**Fig 8:**
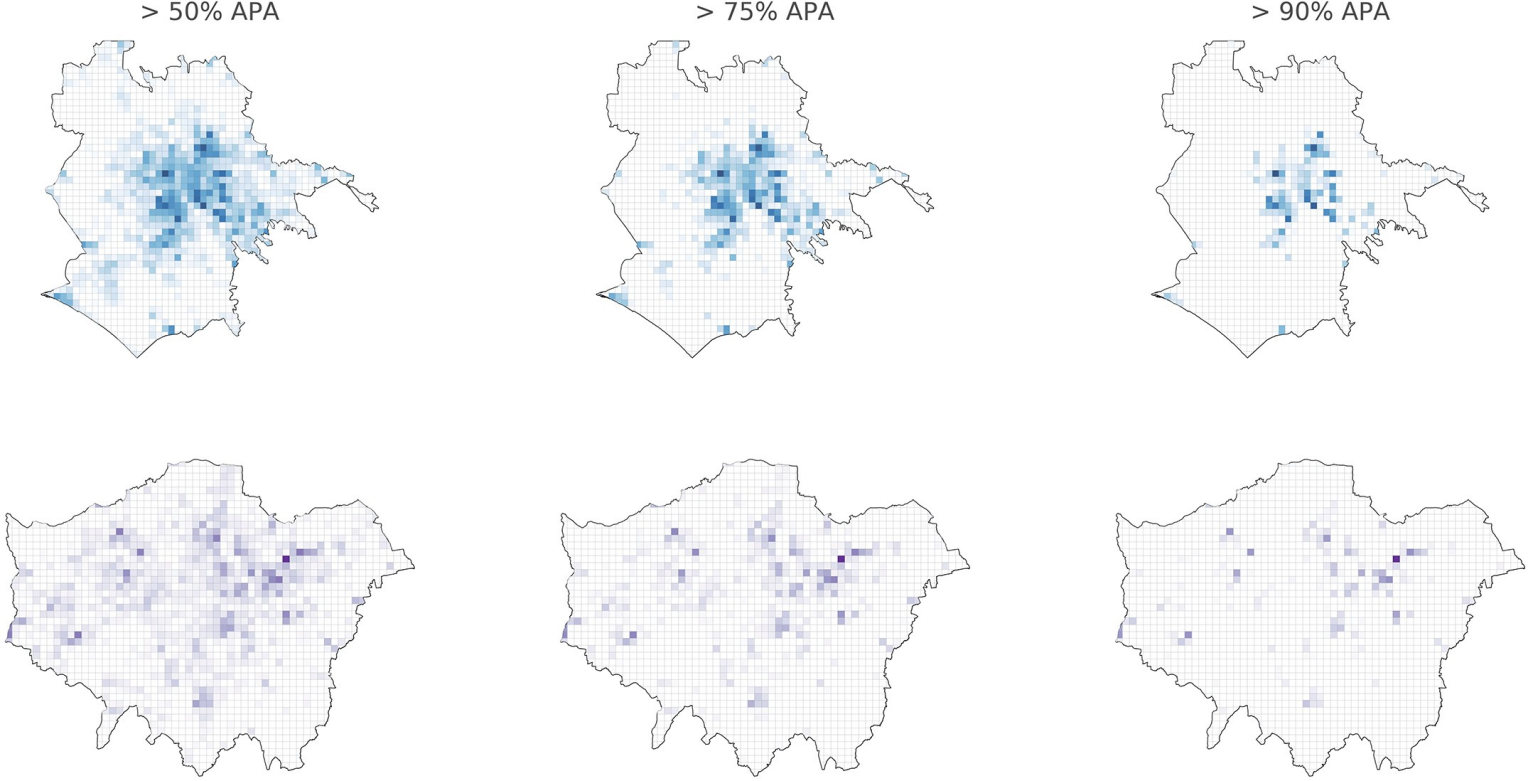
Hotspot locations with APA values greater than the 50th, 75th, and 90th percentiles in (a) Rome and (b) London.

Fig 8 shows “hotspot” locations with APA values greater than the 50th, 75th, and 90th percentiles in Rome (a) and London (b). Remark the differences in “hotspot” locations in both cities for several percentiles. The “hotspot” concentration in Rome is significantly higher than in London, where we see spatial spread.

**Fig 9:**
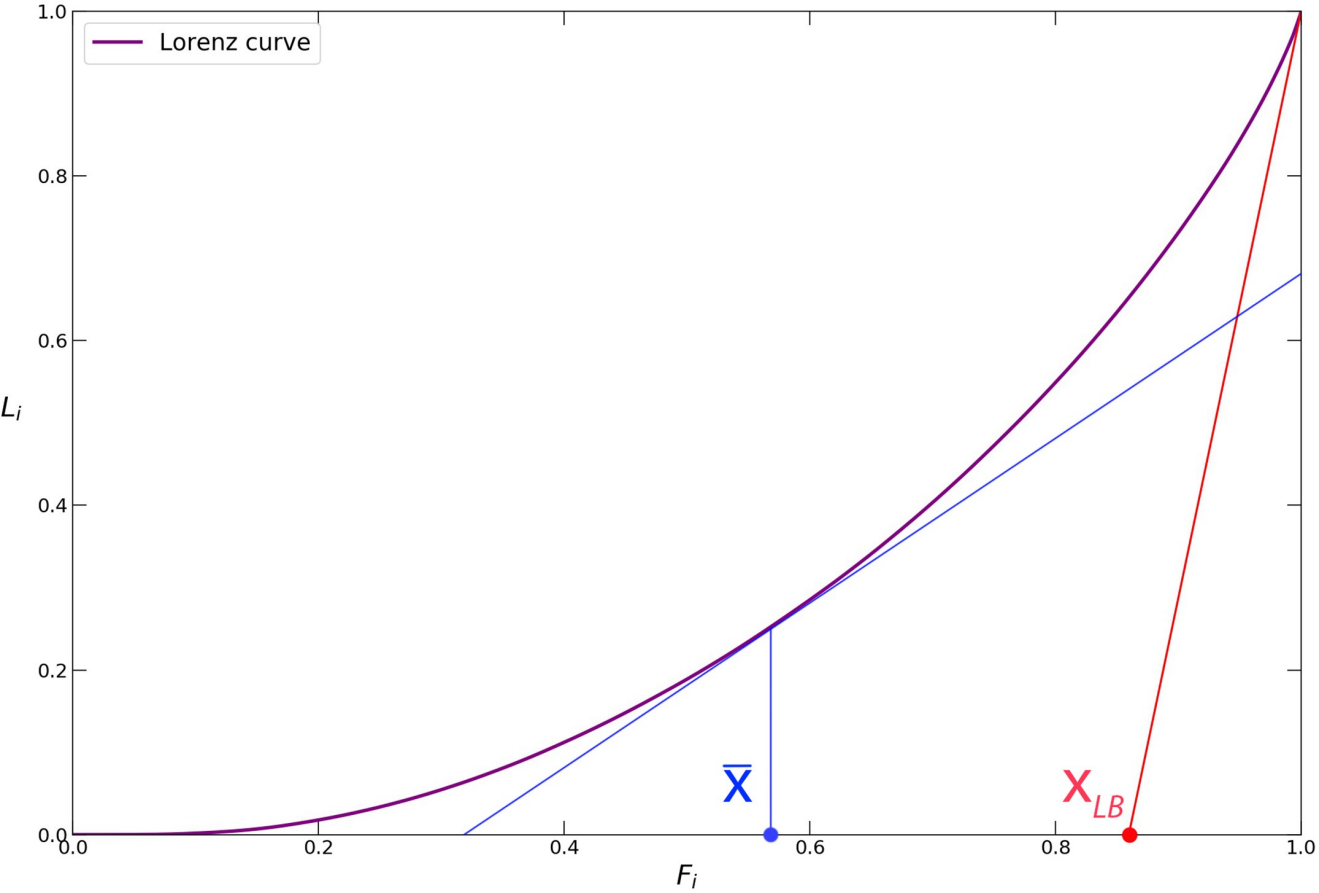
Lorenz curve for a data distribution.

It is essential to perform a meaningful choice of the $\vec{x^{*}}$ for identifying the “hotspots” in Eq (5). With the aim of choosing a threshold which will retain information without turning to noisy behaviour, we resort to a heuristic technique proposed in [40] based on the Lorenz curve from economics, see Fig 9.

For a given distribution of data, the construction of the Lorenz curve proceeds as follows. For a set of values of cardinality *n*, the values are ordered in a non-decreasing sequence $\vec{x_{i}}$ with *i* = 1…*n*. The incomplete sums $L_{i}\equiv (\sum_{j=1}^{i}\vec{x_{j}})/(\sum_{j=1}^{n}\vec{x_{j}})$ are then plotted against *F*_*i*_ ≡ *i*/*n*. As described in [40], we note that the mean value $\vec{\bar{x}}$ corresponds to the projection point of the tangent of slope 1 on the *x*-axis and inverting $F\left(\bar{x}\right)=F_{x}$. The $\vec{x_{LB}}$ value is found from the intersection of the *x*-axis with the tangent of the Lorenz curve at *F*_*i*_ = 1 (red line). This method, called “LouBar”, is inspired by the classical technique for determining the scale for an exponential decay. Indeed, if the decay from *F* = 1 were an exponential exp − (1 − *F*)/*a* where *a* is the scale to be determined, the described method would yield $1-\vec{x_{LB}}=a$.

**Fig 10:**
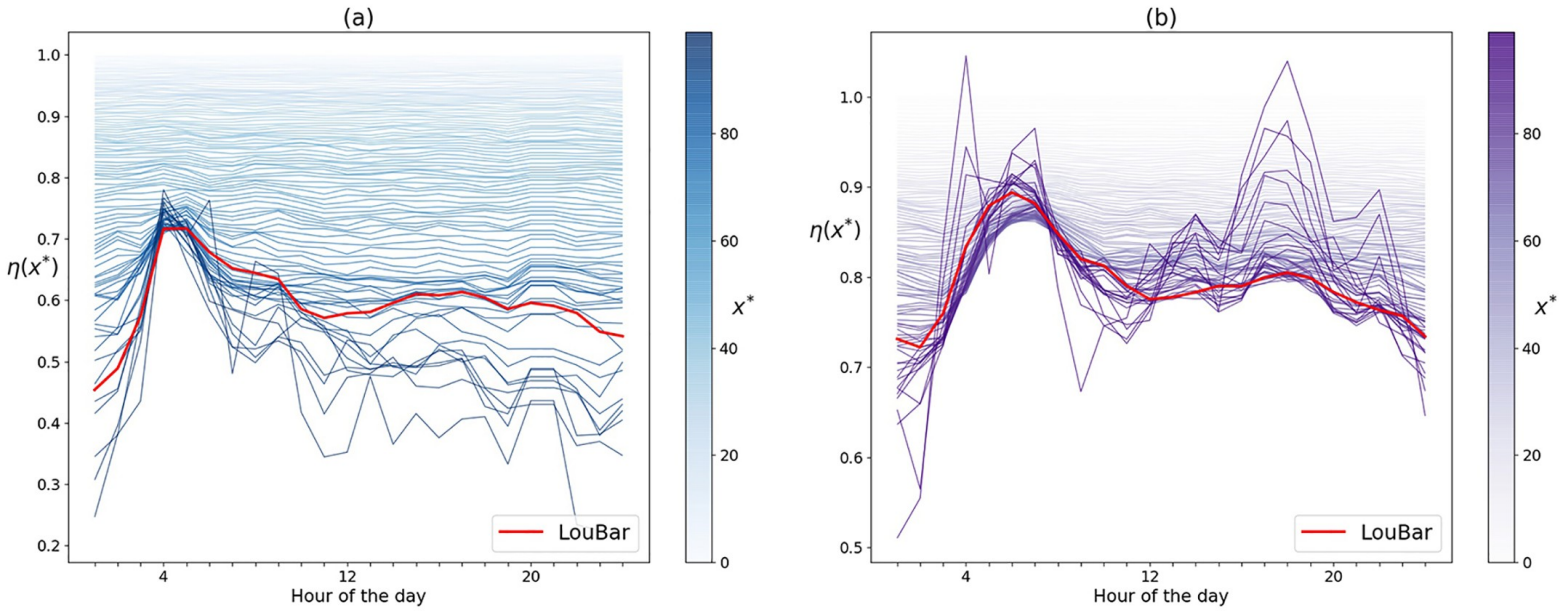
Spreading indices over time for various thresholds ${\vec{x}}^{*}$ in (a) Rome and (b) London.

In Fig 10 we plot the spreading indices for different threshold values $\vec{x^{*}}$ over time in Rome and London. For low values of $\vec{x^{*}}$, the plots show relatively constant, low variance spreading indices over time, while for very large threshold values the spreading indices tend to become noisy.

In fact, the thresholds $\vec{x^{*}}=\vec{\bar{x}}$ and $\vec{x^{*}}=\vec{x_{LB}}$ form an interval $\left[\vec{\bar{x}},\vec{x_{LB}}\right]$ containing all reasonable choices for determining the “hotspots”. However, since the lower bound $\vec{\bar{x}}$ results in a curve with little variation during the day, and since values from the interval closer to the LouBar value give similar results to the Loubar value itself, we will proceed with this choice (see Fig 10).

### Computing the spreading index

In this section, we present the results of studying the spreading index profiles on a typical day in Rome and London, and build hypotheses regarding their interpretations.

**Fig 11:**
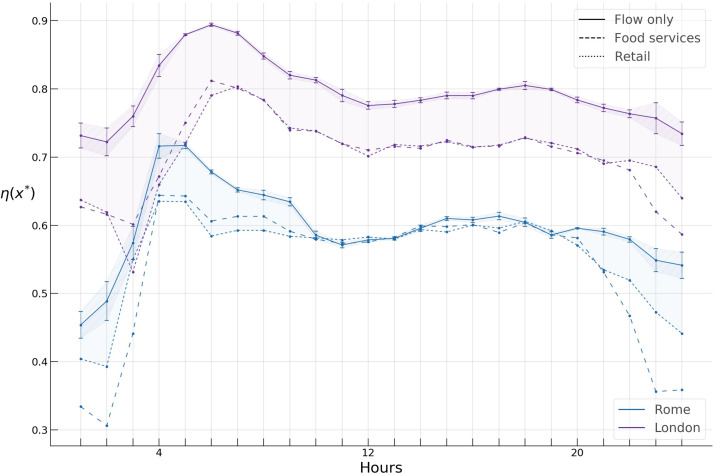
Spreading indices for flow only, food services, and retail activity in Rome and London during a typical day.

Having chosen the threshold value $\vec{x^{*}}$, we compute the hourly profiles of the spreading indices for flows only, food services, and retail activities in Rome and London. Since the data sets of raw GPS trajectories at our disposal span two years, we extract hourly *OD* networks across the working days and obtain sampling distributions and corresponding 95% confidence intervals of spreading indices at each hour with the aim of testing our results for robustness (Fig 11). The wider confidence intervals in the night hours are due to less available data for these hours.

First, we find a significant difference in the *spreading index* hourly profiles of Rome and London. During a typical day, the former varies from around 0.4 to 0.7, while the latter varies from around 0.65 to almost 0.9, suggesting a considerably higher concentration of “hotspots” during the day in Rome compared to London.

Next, we see structural similarities in the hourly patterns of the spreading indices in both cities. As shown in Fig 11, the spreading indices for all types of activities demonstrate a similar inverted U-like pattern, with the spreading index increasing considerably during the night hours, bulging during the morning and evening hours, and declining during the late evening hours. The rapid rise of the index during the night hours could possibly be attributed to the fact that most mobility during these hours is due to flows on highways located in the periphery of both cities, thus yielding a higher *η*, while the bulging of the index at morning and evening hours is likely due to core-periphery commuting flows.

We further observe a large gap of around 0.1 between the flow only *η* profile and those of food services and retail in London, while similar, albeit smaller gaps in Rome can be observed only during the late evening and night hours (shaded areas in Fig 11, whereas the profiles for all types of activities collapse very close to each other during the working hours. This gap can likely be attributed to the “London congestion charge” https://tfl.gov.uk/modes/driving/congestion-charge, which has dramatically reduced private cars in central London since its introduction, while most of food services and retail stores and shops are located in the central part of London, bringing the spreading index down for these activities. In Rome, on the other hand, a similar gap exists only during the night and early morning hours, which one can intuitively expect since most of the food services and shops have a central location, decreasing *η*, while during these hours most of the flows are due to inter-peripheral highway flows which increase *η*.

**Fig 12:**
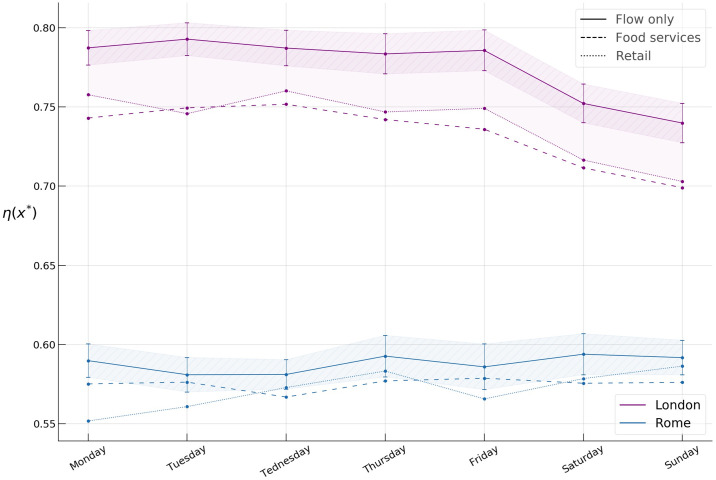
Spreading indices for flow only, food services, and retail activity in Rome and London during the week.

In Fig 12, the spreading indices across the days of the week are shown. We use the 104 weeks of available data to build an empirical 95% confidence interval for the *spreading index*. We see the already familiar gap between the flow only and the other two types of activities in London. Further, we detect a statistically significant (*p* < 1*e*^−5^) change in the index for London, while no significant change appears to be present in Rome.

### The time-space spreading index (TSI)

We have previously computed and tracked the *spreading index* *η* over a typical day in Rome and London. The *spreading index*, being based on Euclidean distances between the cell centroids, represents geographic space, but fails to capture urban mobility. In particular, due to congestion in cities at peak hours, travel times can be said to distort the perception of space. If travel times are considered as a measure of distance, geographically very close locations in the city center might turn out to be further away than geographically further placed locations in the city periphery with low traffic. For this reason, we enable the spreading index to capture urban mobility by introducing the *time-space spreading index* (*TSI*), essentially weighting the distances in the calculation of the spreading index *η* by the pairwise average travel times:

$$\begin{array}{c} TSI({\vec{x}}^{*})=\frac{\frac{1}{N({\vec{x}}^{*})}\sum_{i,j}t\left(i,j\right)1_{\left({\vec{x}}_{i}>{\vec{x}}^{*}\right)}1_{\left({\vec{x}}_{j}>{\vec{x}}^{*}\right)}}{\frac{1}{N}\sum_{i,j}t\left(i,j\right)}, \end{array}$$ (7)

where *t*(*i*, *j*) is the average travel time from cell *i* to cell *j*, and is obtained using the Google Distance Matrix API.

This constitutes an important dimension for studying the spatio-temporal characteristics of the “hotspots” in the mobility networks.

Therefore, we then proceed to analyze the time-space spreading index *TSI* of the three activities during a typical day in Rome and London.

**Fig 13:**
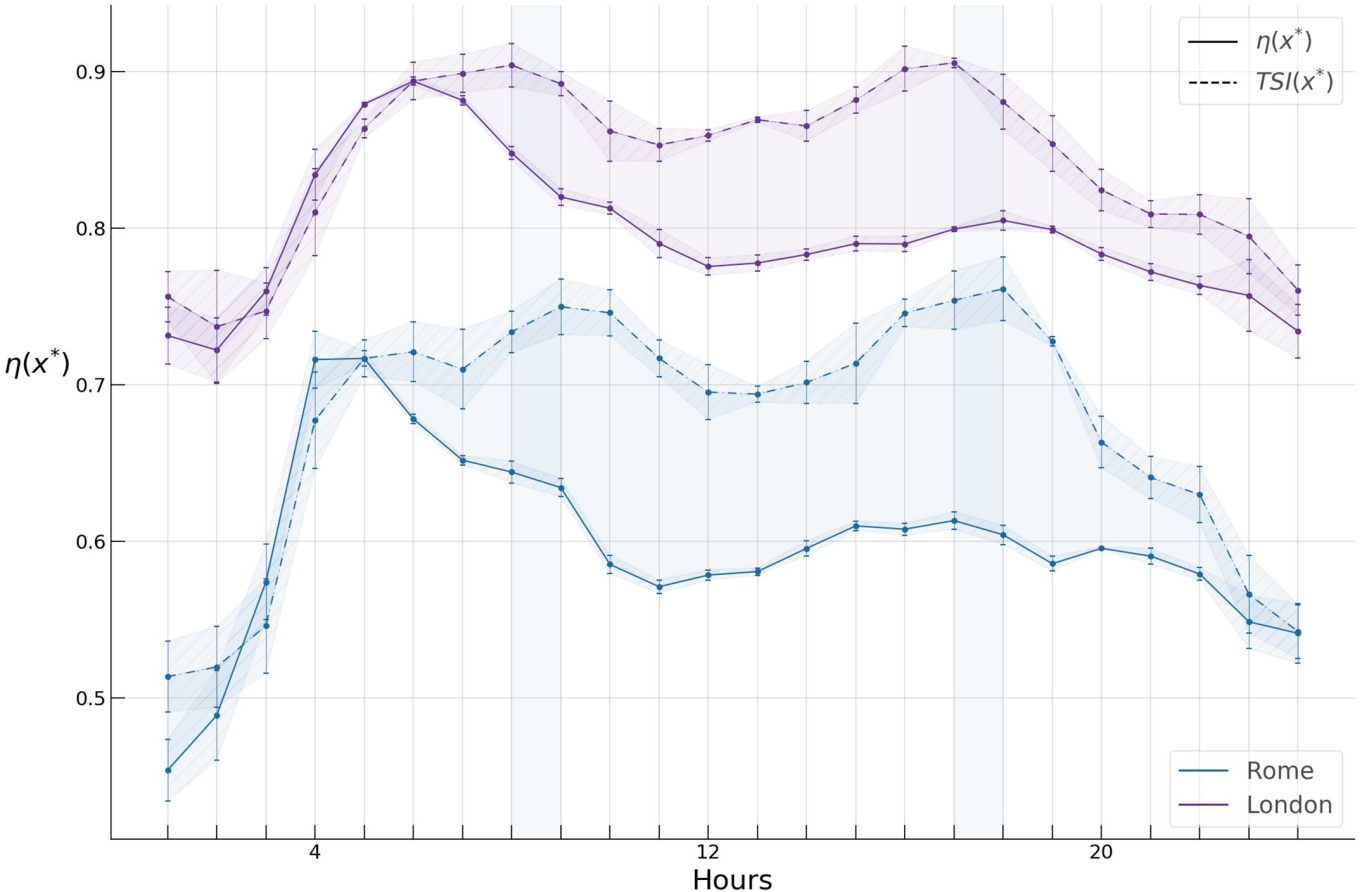
Spreading index and time-space spreading index (*TSI*) with corresponding 95% confidence intervals during a typical day in Rome and London.

**Fig 14:**
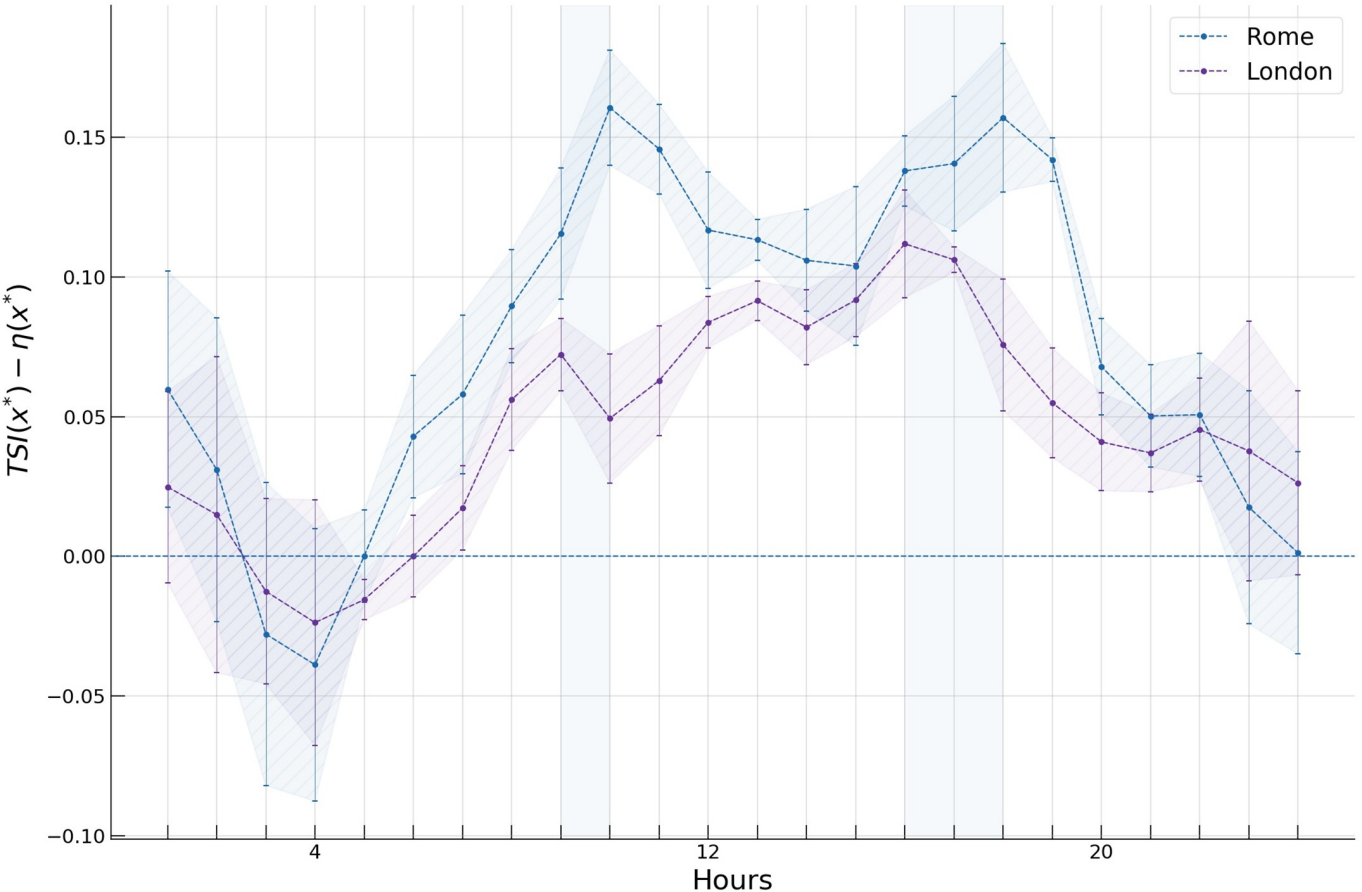
Retail APA values at 18:00 in Rome represented with pairwise time-weighted distances between grid cells using multidimensional scaling (*MDS*). The inset shows the same set of values in geographical space.

The spreading indices and *TSI*s for Rome and London are shown in Fig 13. While the two measures are very close to each other during the night hours, they start to deviate significantly during the rest of the day. At these hours, the *TSI* in both cities is considerably higher than the *spreading index*, hinting at the above-mentioned space-time distortion, in which geographically close central locations become further apart because of longer travel times due to traffic, effectively increasing the *TSI* compared to the *spreading index*. This effect is shown in Fig 14, where the time-weighted distances used in computing the *TSI* are visualised with multidimensional scaling (*MDS*) [55].

Note that the confidence intervals for the *TSI* values are wider than those of the *spreading indices* since additional uncertainty is introduced in the calculation of the *TSI* by including travel times contingent on volatile traffic conditions (Fig 13).

**Fig 15:**
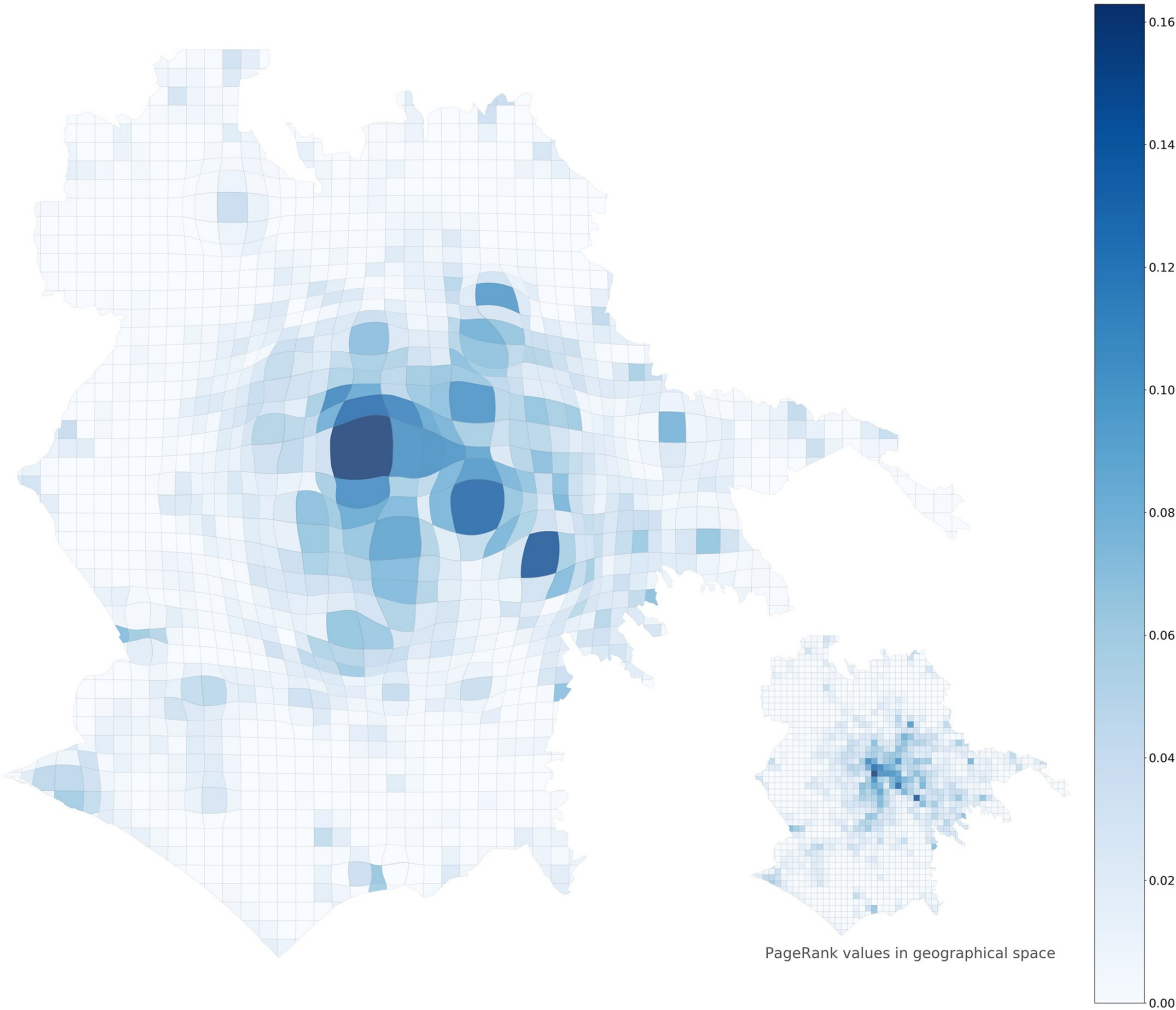
Tracking the difference $TSI\left(\vec{x^{*}}\right)-\eta \left(\vec{x^{*}}\right)$ in Rome and London during a typical day.

We also note the two peaks of higher *TSI* values during the morning and evening commuting hours forming a circadian rhythm in both cities. A peculiar observation is the mismatch of the peaks between the two cities. Rome seems to be “late” by roughly an hour (vertical shaded areas in Figs 13 and 15).

In Fig 15 we plot the differences $TSI\left(\vec{x^{*}}\right)-\eta \left(\vec{x^{*}}\right)$ during the day in Rome and London. We observe this difference during the day to be consistently greater in Rome, suggesting congestion to have a larger impact on the spatio-temporal characteristics of the “hotspots” in Rome.

**Fig 16:**
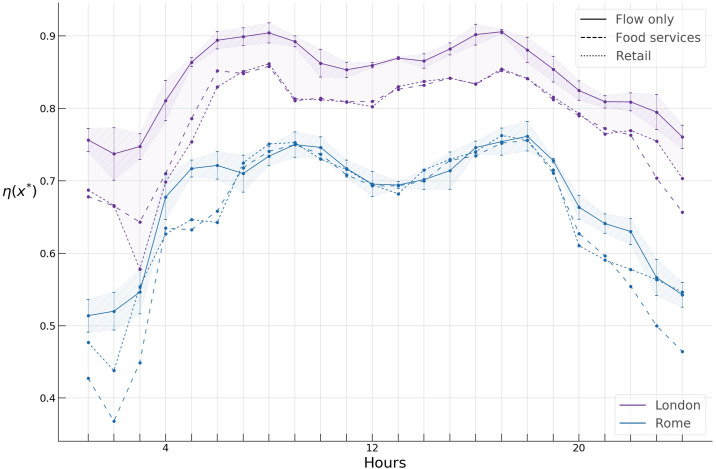
TSI for flow only, food services, and retail activity in Rome and London during a typical day.

The *TSI* for the hotspots of the three types of activities during a typical day in both cities are displayed in Fig 16. One can note a gap in London between the flow only temporal *TSI* profile, and the food services and retail *TSI* profiles, consistent with a similar gap in the case of the spreading index discussed in *Computing the Spreading index* subsection.

## Conclusion

In this paper, we have proposed a generic end-to-end workflow for analyzing spatio-temporal characteristics of urban mobility induced “hotspots” for different types of activities in cities, and have demonstrated it in case studies in Rome and London. The proposed workflow comprised data mining of GPS data, the subdivision of the urban territory into regular grid cells, construction of temporal *OD* networks, addition of socio-economic activity attributes to the *OD* network nodes from *PoI* data, computation of the attribute-enhanced APA centralities in the *OD* networks on an hourly or daily basis, identification of “hotspots”, and visualisation and analysis of measures of their spatial heterogeneity. The obtained results led us to a series of hypotheses regarding their nature, the study of which will be the target of future work.

In particular, we observed an increase in both the *Gini coefficients* as well as the *spreading indices* during the night hours, suggesting higher inequality and spatial spread, respectively. However, a further decomposition of these measures would be required to determine what share of these inequality and spatial spread is due to core-periphery, inter-peripheral, or highway transit flows. Also, future work will be aimed at understanding whether there is a hierarchy of “hotspots” and how it evolves over time. Further, the hypothesis that the peculiar gap between the flow only and food services and retail *spreading index* profiles in London has to do with the congestion charge, and whether our approach can be adopted as a traffic management indicator, requires further study.

Another direction for future work would be the choice of other “hotspot” identification techniques, including that described in [42], and to study the effects of spatial resolution of the grid on their results.

Further yet, we note that a methodology needs to be developed and tested for using the measures proposed in this paper as monitoring tools in connection with specific urban planning policies in a particular city. For instance, deciding critical values of the proposed measures, beyond which action would be required on the part of the urban planners.

Notwithstanding the mentioned shortcomings, our approach has direct utility to urban planners and policy makers. It highlights the road map for creating analysis, visualisation, early warning, or trend detection tools with simple information-rich measures for monitoring city-wide spatial characteristics of mobility related to various socio-economic activities. The proposed workflow from raw data input to analysis and visualisation is generic enough to accommodate other types of spatial movement data (e.g., call detail, smart card, etc.) as well as other socio-economic activities in cities over both short and long terms.

## References

[1] Barthelemy M. Spatial Networks. Physics Reports, 2014; 499: 1–101. 10.1016/j.physrep.2010.11.002

[2] Barthelemy M. The Structure and Dynamics of Cities. Cambridge University Press; 2016.

[3] Bettencourt L. and West G. A unified theory of urban living. Nature, 2010; 467: 912–913. 10.1038/467912a 20962823

[4] Bretagnolle A., Daudé E. and Pumain D. From theory to modelling: urban systems as complex systems. CyberGeo: Eur. J. Geogr, 2006; 335: 1–17.

[5] Lenormand M., Picornell M., Cantú-Ros O.G., Louail T., Herranz R., Barthelemy M., et al Comparing and modelling land use organization in cities. Royal Society open science, 2015; 2(12): 150449 10.1098/rsos.150449 27019730 PMC4807451

[6] Porta S., Strano E., Iacoviello V., Messora R., Latora V., Cardillo A., et al Street centrality and densities of retail and services in Bologna, Italy. Environment and Planning B: Planning and design, 2009; 36(3): 450–465. 10.1068/b34098

[7] Bagrow J.P. and Lin Y.R. Mesoscopic structure and social aspects of human mobility. PloS one, 2012; 7(5): e37676 10.1371/journal.pone.0037676 22701529 PMC3365118

[8] Bingham-Hall J. and Law S. Connected or informed?: Local Twitter networking in a London neighbourhood. Big Data & Society, 2015; 2(2): 2053951715597457.

[9] Botta F. and del Genio C.I. Analysis of the communities of an urban mobile phone network. PloS one, 2017; 12(3): e0174198 10.1371/journal.pone.0174198 28334003 PMC5363903

[10] Rinzivillo S., Mainardi S., Pezzoni F., Coscia M., Pedreschi D. and Giannotti F. Discovering the geographical borders of human mobility. KI-Künstliche Intelligenz, 2012; 26(3): 253–260. 10.1007/s13218-012-0181-8

[11] Zhou Y., Lau B.P.L., Yuen C., Tuncer B. and Wilhelm E. Understanding urban human mobility through crowdsensed data. IEEE Communications Magazine, 2018; 56(11): 52–59. 10.1109/MCOM.2018.1700569

[12] Freeman L.C. Centrality in social networks conceptual clarification. Social networks, 1978 1(3): 215–239. 10.1016/0378-8733(78)90021-7

[13] Bavelas A. Communication patterns in task oriented groups. The Journal of the Acoustical Society of America, 1950; 22(6): 725–730. 10.1121/1.1906679

[14] Freeman L.C. A set of measures of centrality based on betweenness. Sociometry, 1977; 35–41. 10.2307/3033543

[15] Newman M.E. Mathematics of networks. The new Palgrave dictionary of economics, 2016; 1–8.

[16] Pedroche F. Metodos de calculo del vector PageRank (in spanish). Bolletin Sociedad Espanola Matematica Aplicada, 2007; 39: 7–30.

[17] Agryzkov T., Oliver J.L., Tortosa L. and Vicent J.F. An algorithm for ranking the nodes of an urban network based on concept of PageRank vector. Applied Mathematics and Computation, 2012; 219: 2186–2193. 10.1016/j.amc.2012.08.064

[18] Agryzkov T., Oliver J.L., Tortosa L. and Vicent J.F. New highlights and a new centrality measure based on the Adapted PageRank Algorithm for urban networks. Applied Mathematics and Computation, 2016; 291: 12–29. 10.1016/j.amc.2016.06.036

[19] Benyahia O. and Largeron C. Centrality for graphs with numerical attributes. IEEE/ACM International Conference on Advances in Social Networks Analysis and Mining (ASONAM), 2015; 1348-1353.

[20] Holme P. and Saramäki J. Temporal networks. Physics reports, 2012; 519(3): 97–125. 10.1016/j.physrep.2012.03.001

[21] Ramachandra T.V., Aithal B.H. and Sanna D.D. Insights to urban dynamics through landscape spatial pattern analysis. International Journal of Applied Earth Observation and Geoinformation, 2012; 18: 329–343. 10.1016/j.jag.2012.03.005

[22] Crucitti P., Latora V. and Porta S. The network analysis of urban streets: a primal approach. Planning and design, 2006; 33(5): 705–725. 10.1068/b32045

[23] Saberi M., Mahmassani H.S., Brockmann D. and Hosseini A. A complex network perspective for characterizing urban travel demand patterns: graph theoretical analysis of large-scale origin–destination demand networks. Transportation, 2017; 44(6): 1383–1402. 10.1007/s11116-016-9706-6

[24] Yildirimoglu M. and Kim J. Identification of communities in urban mobility networks using multi-layer graphs of network traffic. Transportation Research Part C: Emerging Technologies, 2018; 89: 254–267. 10.1016/j.trc.2018.02.015

[25] Crucitti P., Latora V. and Porta S. The network analysis of urban streets: a dual approach. Physica A: Statistical Mechanics and its Applications, 2006; 369(2): 853–866. 10.1016/j.physa.2005.12.063

[26] Bonaventura M., Aiello L.M., Quercia D. and Latora V. Predicting Urban Innovation from the Workforce Mobility Network in US. arXiv preprint, 2019; arXiv:1911.00436.

[27] Kirkley A., Barbosa H., Barthelemy M. and Ghoshal G. From the betweenness centrality in street networks to structural invariants in random planar graphs. Nature communications, 2018; 9(1): 2501 10.1038/s41467-018-04978-z 29950619 PMC6021391

[28] De Montis A., Barthélemy M., Chessa A. and Vespignani A. The structure of interurban traffic: a weighted network analysis. Environment and Planning B: Planning and Design, 2007; 34(5): 905–924. 10.1068/b32128

[29] Wen T.H. Geographically modified PageRank algorithms: Identifying the spatial concentration of human movement in a geospatial network. PloS one, 2015; 10(10): e0139509 10.1371/journal.pone.0139509 26437000 PMC4593571

[30] Nie K., Wang Z., Du Q., Ren F. and Tian Q. A network-constrained integrated method for detecting spatial cluster and risk location of traffic crash: A case study from Wuhan, China. Sustainability, 2015; 7: 2662–2677. 10.3390/su7032662

[31] Yamada I. and Thill J.-C. Comparison of planar and network k-functions in traffic accident analysis. Journal of Transport Geography, 2004; 12: 149–158. 10.1016/j.jtrangeo.2003.10.006

[32] Tiwari N., Adhikari C., Tewari A. and Kandpal V. Investigation of geo-spatial hotspots for the occurrence of tuberculosis in almora district, India, using GIS and spatial scan statistic. International Journal of Health Geographics, 2006; 5: 33 10.1186/1476-072X-5-33 16901341 PMC1557839

[33] Zhao P., Qin K., Ye X. and Wang Y.A. Trajectory clustering approach based on decision graph and data field for detecting hotspots. International Journal of Geographical Information Science, 2017; 31(6): 1–27.

[34] Zheng Y., Zhang L.Z. and Xie X. Mining interesting locations and travel sequences from GPS trajectories. Proceedings of the 18th International Conference on World Wide Web, April 2009, Madrid, Spain, 791–800.

[35] Ahas R., Aasa A., Silm S. and Tiru M. Daily rhythms of suburban commuters movements in the Tallinn metropolitan area: case study with mobile positioning data. Transportation Research Part C: Emerging Technologies, 2010; 18(1): 45–54. 10.1016/j.trc.2009.04.011

[36] Mao F., Minhe J. I. and Liu T. Mining spatiotemporal patterns of urban dwellers from taxi trajectory data. Frontiers of Earth Science, 2016; 10(2): 205–221. 10.1007/s11707-015-0525-4

[37] Anselin L. Local indicators of spatial association—Lisa. Geographical Analysis, 2004; 27: 93–115. 10.1111/j.1538-4632.1995.tb00338.x

[38] Hu Y., Miller H.J. and Li X. Detecting and analysing mobility hotspots using surface networks. Transactions in GIS, 2014; 18: 911–935. 10.1111/tgis.12076

[39] Lichman M. and Smyth P. Modelling human location data with mixtures of kernel densities. Proceedings of the 20th ACM SIGKDD International Conference on Knowledge Discovery and Data Mining, August 2014, New York, USA, 2014; 35–44.

[40] Louail T., Lenormand M., Ros O.G.C., Picornell M., Herranz R., Frias-Martinez E., et al From mobile phone data to the spatial structure of cities. Scientific reports, 2014; 4: 5276 10.1038/srep05276 24923248 PMC4055889

[41] Louail T., Lenormand M., Picornell M., Cantu O.G., Herranz R., Frias-Martinez E., et al Uncovering the spatial structure of mobility networks. Nature Communications, 2015; 6(1): 6007 10.1038/ncomms7007 25607690

[42] Hamedmoghadam H., Ramezani M., and Saberi M. Revealing latent characteristics of mobility networks with coarse-graining. Scientific reports, 2019; 9: 7545 10.1038/s41598-019-44005-9 31101843 PMC6525175

[43] Wang X., Zhou Z., Xiao F., Xing K., Yang Z., Liu Y., et al. Spatio-temporal analysis and prediction of cellular traffic in metropolis. 2017 IEEE 25th International Conference on Network Protocols (ICNP), Oct. 2017; 1-10.

[44] Vlahogianni E. Computational intelligence and optimization for transportation big data: challenges and opportunities. Engineering and Applied Sciences Optimization, 2015; 107–128. 10.1007/978-3-319-18320-6_7

[45] Chen Q., Song X., Yamada H. and Shibasaki R. Learning deep representation from big and heterogeneous data for traffic accident inference. Proceedings of the Thirtieth AAAI Conference on Artificial Intelligence, 2016; 338–344.

[46] Jia Y., Wu J. and Du Y. Traffic speed prediction using deep learning method. Proceedings of 19th International Conference on Intelligent Transportation Systems (ITSC) IEEE, 2016; 217–1222.

[47] Page L., Brin, Motwani R. and Winogrand T. The pagerank citation ranking: Bringing order to the web. Technical report 1999-66, Stanford InfoLab, 1999, Stanford, CA, USA.

[48] Berkin P. A survey on PageRank computing. Internet Mathematics, 2005; 2(1); 73–120. 10.1080/15427951.2005.10129098

[49] Bianchini M., Gori M. and Scarselli F. Inside PageRank. ACM Transactions on Internet Technology, 2005; 5(1): 92–128. 10.1145/1052934.1052938

[50] Rey S.J. and Smith R.J. A spatial decomposition of the Gini coefficient. Letters in Spatial and Resource Sciences, 2013; 6(2): 55–70 10.1007/s12076-012-0086-z

[51] Volpati V. and Barthelemy M. The spatial organization of the population density in cities. arXiv preprint, 2018; arXiv:1804.00855.

[52] Fontanari A., Taleb N.N. and Cirillo P. Gini estimation under infinite variance. Physica A: Statistical Mechanics and its Applications, 2017; 502: 256–269. 10.1016/j.physa.2018.02.102

[53] OpenStreetMap 2019. https://www.openstreetmap.org. [Accessed September 2019].

[54] Alstott J., Bullmore E. and Plenz D. Powerlaw: a Python package for analysis of heavy-tailed distributions. PloS one, 2014; 9(1): e85777 10.1371/journal.pone.0085777 24489671 PMC3906378

[55] Ahmed N. and Miller H.J. Time–space transformations of geographic space for exploring, analyzing and visualizing transportation systems. Journal of Transport Geography, 2007; 15(1): 2–17. 10.1016/j.jtrangeo.2005.11.004

